# Treatment of an 4.6 renal pelvic stone with staged ureteroscopy using a C-VAC device OR: How I learned to stop worrying and sit during ureteroscopy

**DOI:** 10.51894/001c.144646

**Published:** 2025-09-30

**Authors:** Timothy Smith

**Affiliations:** 1 College of Osteopathic Medicine Michigan State University; 2 Department of Urology Detroit Medical Center

## 124

### INTRODUCTION

Suction ureteroscopic devices are changing how we treat nephrolithiasis. Large renal stones of 4 cm or more, and those with infective etiologies are typically reserved for Percutaneous nephrolithotomy (PCNL) or even pyelolithotomy. We present A case of a 4.6 cm calcified fungus ball treated with staged ureteroscopy (URS) using a CVAC (Calyxo) Suction ureteroscopy device (SUD).

### CASE

Patient is a 78 yo male who was found to have a 4.6 cm partially calcified fungal ball in the right renal pelvis. Options including PCNL vs staged URS were discussed. He refused PCNL, and elected for staged URS with the acknowledgement that it could take multiple procedures. He underwent URS 3 total times. The first was without the CVAC device due to challenges with prior authorization for payer coverage, however the subsequent two attempts were with a CVAC. Operative time for the cases were: 111, 79, and 170 minutes respectively. Figure 1 shows the progression of the stone before and after each case. At the end of the third case, there were no stone elements able to be visualized in the entire collecting system. There were no postoperative fevers, infections, or readmissions.

### CONCLUSION

With the incorporation of SUD such as the CVAC, we can treat nephrolithiasis in sized not thought to be possible with staged ureteroscopy. More so, we present a case of treatment of a stone with an infective etiology using a CVAC device. We propose that it is possible to treat large stones typically reserved for PCNL safely and economically with SUD.

